# VineSens: An Eco-Smart Decision-Support Viticulture System

**DOI:** 10.3390/s17030465

**Published:** 2017-02-25

**Authors:** Josman P. Pérez-Expósito, Tiago M. Fernández-Caramés, Paula Fraga-Lamas, Luis Castedo

**Affiliations:** Department of Electronics and Systems, Faculty of Computer Science, Universidade da Coruña, 15071 A Coruña, Spain; josman.perez@udc.es (J.P.P.-E.); paula.fraga@udc.es (P.F.-L.); luis.castedo@udc.es (L.C.)

**Keywords:** wireless sensor networks, IoT, plant diseases and pests, grapevine, vineyards, environmental impact, ecologically sustainable vineyards, smart viticulture, precision agriculture

## Abstract

This article presents VineSens, a hardware and software platform for supporting the decision-making of the vine grower. VineSens is based on a wireless sensor network system composed by autonomous and self-powered nodes that are deployed throughout a vineyard. Such nodes include sensors that allow us to obtain detailed knowledge on different viticulture processes. Thanks to the use of epidemiological models, VineSens is able to propose a custom control plan to prevent diseases like one of the most feared by vine growers: downy mildew. VineSens generates alerts that warn farmers about the measures that have to be taken and stores the historical weather data collected from different spots of the vineyard. Such data can then be accessed through a user-friendly web-based interface that can be accessed through the Internet by using desktop or mobile devices. VineSens was deployed at the beginning in 2016 in a vineyard in the Ribeira Sacra area (Galicia, Spain) and, since then, its hardware and software have been tested to prevent the development of downy mildew, showing during its first season that the system can led to substantial savings, to decrease the amount of phytosanitary products applied, and, as a consequence, to obtain a more ecologically sustainable and healthy wine.

## 1. Introduction

Over the last decades, traditional viticulture or vine-growing has experienced a revolution due to the advent of more environmentally friendly alternatives. Thus, integrated viticulture requires fewer chemicals, ecologic viticulture substitutes synthetic chemicals with products compatible with ecologic agriculture, and bio-dynamic viticulture makes use of bio-dynamic preparations that are applied according to a bio-dynamic schedule. The latest revolution in viticulture is happening thanks to the advances in technology and communications, which are harnessed by precision viticulture to produce high volumes of a given type of grapevine with the quality selected by an oenologist.

Precision viticulture helps farmers to monitor and control the micro-climatic conditions of the vineyard and the diseases and pests that affect the plants. There are different environmental factors and climatic events that affect the state of the soil and the plants. Temperature and Relative Humidity (RH) influence the development of the vineyard (i.e., its growth and maturation) and its diseases. Rain impacts the development of diseases derived from fungi or apoplexy. Hail and wind can cause severe damage to leaves, to the bark, and to the fruit. Soil temperature affects the development of biological processes and the growth of the roots, while soil moisture helps to determine the water needs of the crop. Moreover, frost [1] can pose a serious problem for vine growers, since it can kill the plant tissues and, thus, harm the leaves and fruit formation.

Vines (*vitis vinifera*) are also exposed to different types of pests and diseases, which depend essentially on the area of cultivation. In Europe, where VineSens was devised, the main diseases are grape phylloxera (*Daktulosphaira vitifoliae*) [2], powdery mildew (*Erysiphe necator*, also known as *Uncinula necator*) [3], downy mildew (*Plasmopara viticola*) [4], coulure [5], *Botrytis cinerea* [6], black rot (*Guignardia bidwellii*) [7], and excoriose (*Phomopsis viticola*) [8].

VineSens is focused on the prevention of downy mildew, which is a disease caused by fungus-like microorganisms (oomycetes). Its spores, when placed on the leaves, penetrate through the stomata and colonize the crop. It is one of the most damaging grapevine diseases. When environmental conditions are favorable, it can attack all the organs of a green vine, causing losses of more than 50% of the crop [9]. It appears in regions where the climate is hot and humid during the vegetative growth.

Downy mildew is now endemic to almost all the world’s vineyards and is generally recognized as being destructive on a world-wide basis [10]. Even though there are many effective phytosanitary treatments for controlling grapevine diseases, the most widely used for downy mildew is copper sulphate, which can have a negative environmental impact. It is important to note that vine growers should know at what point in the life cycle of the vine they should take the appropriate measures on their crops and, therefore, it is critical to apply the right treatment, at the right time, and at the right place.

Despite the wine industry’s reputation of being environmentally safe, prior research [11] has shown that the cultivation of wine grapes and the production of wine is associated with a large number of environmental issues. The main concerns include water use and its quality, the production and management of organic and inorganic solid waste, energy use and the generation of Greenhouse Gas (GHG) emissions, the use and management of chemicals in the vineyard and winery, land use issues, and the impact on ecosystems. According to Gerling [10], grapevine cultivation relies heavily upon the use of chemical fungicides: most vine growers apply between 6–10 fungicide sprayings per season. For example, in France the cost of fungicides for powdery mildew control is around 75 million euros per year and it does not consider the increasing fuel costs associated with the application of such fungicides.

The objective of VineSens is to create eco-smart vineyards supported by an integrated technological system. Such a system is able to monitor a vineyard in real-time and offers a web-based tool that provides up-to-date information on the state of the vines. Furthermore, VineSens supports the decision-making of the vine grower. The system notifies when downy mildew has reached a threshold when preventive measures have to be taken. Thus, VineSens focuses on reducing the use of chemicals by providing the vine grower with a custom plan to control the disease, which leads to a more ecologic and sustainable solution. The system also delivers lower operating costs by using weather and disease indexes to reduce labor and the number of spray cycles.

In addition, the monitoring system provides the historical and real-time values of different relevant environmental parameters, generating statistics that may help to take specific measures to improve the treatments performed. Besides, it helps to predict the consequences of climate-driven changes, as pests and their host plants are interdependent.

Finally, it should be emphasized that, although the system focuses on preventing downy mildew, it can be easily extended to monitor other vineyard diseases or to collect data on other relevant parameters.

The remainder of this paper is organized as follows. Section 2 reviews the most relevant academic and commercial systems to monitor vineyards. Section 3 introduces the main epidemiological models for preventing downy mildew. Section 4 details the hardware and software components of VineSens. Section 5 describes the experimental setup and results of different tests performed on VineSens. Finally, Section 6 is devoted to the conclusions.

## 2. Related Work: Academic and Commercial Systems for Vineyard Monitoring

Over the last years, different researchers and companies have proposed solutions for monitoring the state of a vineyard by using sensors. Such sensors, when deployed throughout a vineyard, allow us to distinguish the specific necessities of each area, what improves the management of the vineyard and addresses the possible problems accurately.

In the literature, different techniques for monitoring the health of a vineyard can be found. For instance, vineyard status can be analyzed through the vine architecture (canopy zone analysis). The continuous evaluation of the canopy volume and the tree area index in vineyards is one of the main objectives in precision viticulture. The Leaf Area Index (LAI), defined as the one-side leaf area per unit ground area, is a widely used index to characterize grapevine vigor. For example, in [12] the authors proposed the use of a laser sensor for monitoring within-field leaf area variability. The authors use LIDAR (Light Detection and Ranging) techniques to estimate LAI and canopy density in grapevines. The main difficulties that arise when using this technology are the non-random distribution of leaves and the presence of other non-green vegetative elements within the canopy.

Most of the recent literature focuses on remote monitoring grapevines by using thermal imaging (aerial and ground-based) and hyperspectral techniques [13,14,15,16,17]. These remote sensing approaches involve the acquisition of spectral data from expensive platforms, such as satellites, aircrafts, or Remotely Piloted Aerial Systems (RPAS).

Other authors monitor vineyards by collecting agrochemical data. Such data can be used to improve the production process and to guarantee traceability and accurate grapevine health observations. UHF (Ultra-High Frequency) and LF (Low-Frequency) transponders are able to link data (e.g., treatments) to geolocated plants. For example, in [18] the authors evaluate the impact of specifically designed UHF and LF transponders when implanted in grapevine rootstocks. Although the traceability information is useful, the authors conclude that the implantation procedures affect the growth of the shoots analyzed.

Decision Support Systems (DSS) are also described in the literature. For example, *Vite.net*, an holistic approach for the sustainable management of vineyards, is presented in [19]. Its user-friendly DSS includes a real-time monitoring system and a web-based tool that analyzes the data collected by using advanced modeling techniques, providing up-to-date information for managing the vineyard. The system’s aim is to assist the decision maker rather than replacing it. The article is an excellent reference on how to implement a software monitoring system and shows promising results obtained in Italian vineyards. However, the authors rely on third-party hardware, so the details on the sensor nodes are omitted.

Wireless Sensor Networks (WSN) based on the Wi-Fi standards (IEEE 802.11 a/b/g/n), Bluetooth, and ZigBee have been used extensively in different fields for monitoring different parameters [20], appliances [21,22], military resources [23,24,25,26], physical locations [27], equipment [28], and even musical instruments [29]. An example of an WSN-based monitoring system that makes use of IEEE 802.11 a/b/g/n transceivers is presented in [30]. In such a system each sensor node takes pictures of a vineyard and uses image processing techniques to detect unusual events on the leaves. The main drawback of the system is that is not able to distinguish between deficiency, pest, disease or other harmful agents. Nevertheless, the authors suggest creating a symptoms image database and a neural network to provide an accurate problem diagnosis.

NAV [31] is a WSN for remote monitoring vineyards in real-time. The system includes a weather station and several wireless nodes located in the vineyard. A GSM modem installed on the nodes transfers the data to a remote central server. NAV collects exclusively micro-meteorological parameters without considering disease prevention.

A ZigBee WSN-based irrigation DSS for viticulture in protected areas is proposed in [32]. The system monitors parameters such as temperature and soil moisture, and calculates irrigation based on the field data and on rule-based knowledge to make the best decision. Another ZigBee-based solution is detailed in [33], where the system proposed is able to harvest kinetic energy from the wind and from the water that runs through the farm pipes. Additionally, it is worth mentioning the research described in [34], which presents an open-source web-based system that integrates spatial information from remote sensing images, GPS measurements, and inventory data.

Regarding commercial developments, a number of companies currently offer monitoring solutions for vineyards. For instance, VintiOS [35] is a precision agriculture software that supports the decisions of vine growers and oenologists on the grapevine production and its quality. The software shows the location of the farm and allows the user to manage all the data related to it. A similar tool is Monet [36], which monitors the health state of a vineyard, including the risk of developing certain diseases, weather information, and other relevant events. Other proprietary solutions have been developed by Save [37], Ranchsystems [38], and SmartVineyard [39].

After analyzing all the systems mentioned, it can be stated that VineSens presents five main advantages that, as of writing, have not been found together in the academic literature or in commercial developments. First, unlike other systems, it provides a complete system, both hardware and software, so compatibility issues are minimized. Furthermore, this kind of development avoids collecting data from or through third-party (and usually expensive) cloud-based platforms. Second, it uses non-proprietary communications technologies (Wi-Fi), which eases the integration of both VineSens nodes and third-party sensing solutions. Third, thanks also to the use of standard Wi-Fi transceivers, the deployment cost of the system is fairly inexpensive, whilst providing a good coverage area. Fourth, it provides an API for developers in order to further enhance the system, being really simple to create plugins or mobile apps based on the data collected by VineSens. Finally, the fifth advantage is that it has been designed in a modular way, so it is easy to add new epidemiological models, alarms, sensors, and actuators to the system.

## 3. Epidemiological Models for Preventing Downy Mildew

The prevention of downy mildew is related to the study of the stages of its life cycle and to the creation of what is known as an epidemiological model. Thus, the detection of downy mildew has been approached through different empirical and mechanistic models [9]. Most models have been developed to manage a fungicidal programming focused specifically on primary infections. However, note that the primary downy mildew inoculum plays a key role in epidemics not only at the beginning of the growing season, but also in the secondary cycles that follow the first infection in late spring and summer [4].

The existence of relationships between environmental factors, host susceptibility, and the pathogen, have been known for a long time, allowing researchers to create different epidemiological models like the four described briefly in the next subsections: the Rule 3-10, the EPI model, the DMCast model, and the UCSC model.

### 3.1. Rule 3-10

This empirical rule was developed by Baldacci in northern Italy [40]. The rule is called 3-10 because it assumes that primary infections are likely to happen when the following conditions occur simultaneously: air temperature is equal or higher than 10 °C, vine shoots are at least 10 cm long, and at least 10 mm of continuous rain has fallen during the previous 24–48 h.

Once the infection starts, its progress is calculated using the Goidanich model (in Table 1), which shows the percentage of disease development depending on the temperature and on the relative humidity (from 25 °C the development is constant). Note that this model does not take oospore maturing into account and does not distinguish the different stages of the infection process.

### 3.2. EPI Model

The EPI model (Modéle d’état potentiel d’infection) was developed in the region of Bordeaux (France) by Strizyk [41]. It is based on the assumption that the pseudo-fungus *Plasmopara viticola* is adapted to the climatic conditions of a specific area, so climatic variations affect the development of the pathogen directly.

The EPI index represents the pathogen potential infection, which, according to the model, is affected by the current and historical monthly rainfall and temperature, the number of days with rain per month, the rainfall during the last ten days, the average monthly relative humidity at night, and the average daily and monthly temperature.

### 3.3. DMCast Model

DMCast (Downy Mildew foreCast) is a meteorological model designed by Park et al. in Geneva, New York (USA), to provide an estimate of both primary and secondary infections of downy mildew [42].

The model indicates the occurrence of primary infections when there are mature oospores and when weather conditions favor the germination of the oospores, the dispersion of the inoculum, and infections. The model assumes that the dynamics of the maturation of the oospores follow a normal density function and that rain plays a key role in such maturation process. Thus, the model considers as relevant factors the daily rainfall, the average monthly rainfall for a period of 30 years, and the number of rainy days per month.

### 3.4. UCSC Model

The UCSC (Università Cattólica del Sacro Cuore) model [43] is a mechanistic model developed according to the principles of the system analysis proposed by Rabbinge and De Wit in 1989 [44]. The model simulates, with a time step of one hour, the infection process, including the maturation and germination of oospores, the discharge and dispersal of the zoospores (a zoospore is basically an asexual spore provided with motile flagella for locomotion), and the final success of the infection and the subsequent appearance of the disease symptoms.

A detailed explanation of the mathematics behind the model is out of the scope of the article, but the interested reader can find further information in [43,45,46,47].

### 3.5. Model Comparison

A summary of the main characteristics of the different epidemiological models analyzed for preventing downy mildew is shown in Table 2. In order to select the epidemiological model to implement, it must first be noted that some models, like EPI and DMCast, underestimate the risk of infection. Moreover, although the DMCast model gives a good balance between complexity and performance, it is necessary to have a record of the previous 30 years of the average monthly rainfall. There are many locations for which such data are available, but, in the area where VineSens was deployed, the nearest weather station was 12 km from the farm, so data may not be accurate enough. Regarding the UCSC model, it does not need calibration, but it is far more complex than the empirical approaches.

In this article, we only describe the implementation associated with the empirical Rule 3-10 model. Despite being simple, it actually provides a good reference for the development of downy mildew oospores and, therefore, it is possible to prevent actual infections. As a consequence, vine growers currently continue to follow the simple and widely known Rule 3-10 for deciding their spraying schedule. Finally, note that the four models cited in this paper are complementary: they could be applied together to obtain a better prediction of the state and evolution of a downy mildew infection in the vineyard.

## 4. VineSens Design

### 4.1. Functionality of the System

**Figure 1:**
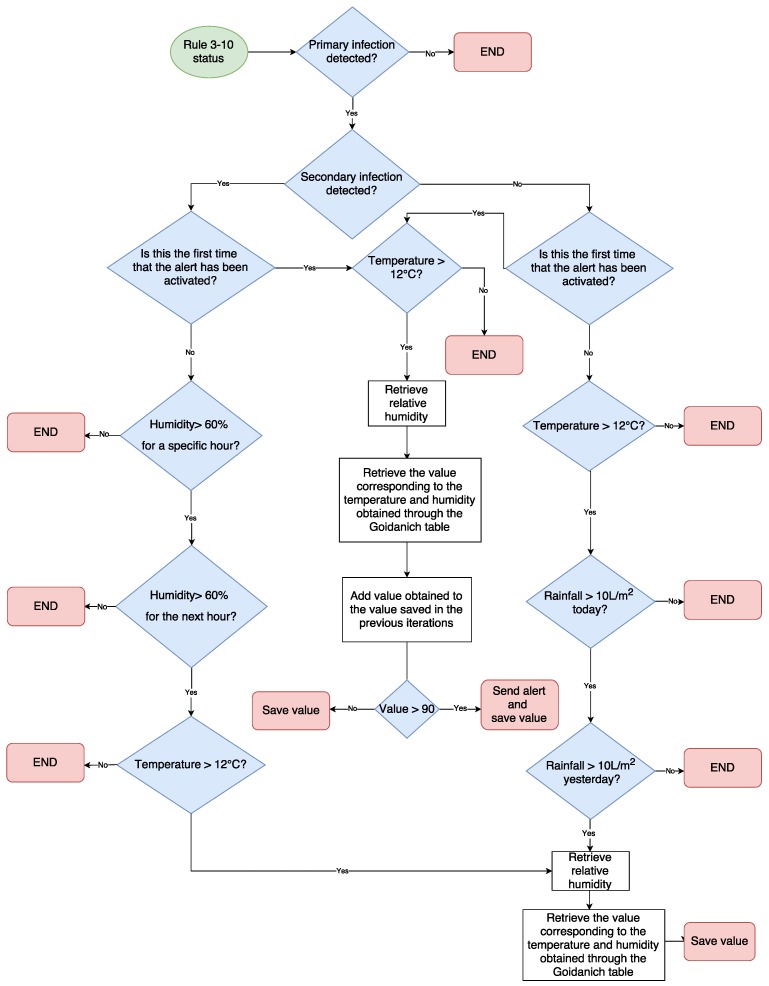
Flow diagram of the Rule 3-10 Algorithm.

VineSens was designed with the objective of providing the vine grower with a simple tool focused on disease prevention for managing a vineyard in a sustainable way. The system was also conceived for helping to monitor the evolution of the vineyard and to increase the quality of grapes, presenting information about parameters and recommendations to support decision-making. Thus, VineSens covers three basic needs:

- It keeps a record of the main parameters that affect the development of the vine, being able to consult them through the Internet by using a wide range of devices.
- It assists in preventing diseases through predictive models, obtaining ad-hoc alerts from the farm. Specifically, a software algorithm based on the Rule 3-10 was devised and implemented. The algorithm automates the detection of both primary and secondary downy mildew infections and sends alerts to the users. Such an algorithm is hosted on a central server that once a day checks the status of the infection using the environmental parameters collected from the nodes. Then, the Rule 3-10 index is calculated according to Table 1. When the accumulated index value exceeds a threshold (e.g., 80%), an alert is sent to the user. A complete flow diagram of the algorithm that illustrates its inner workings and complexity is depicted in Figure 1.
- It helps to reduce and prevent environmental pollution, rationalizing the application of treatments to avoid over-treatment and the presence of harmful environmental agents.

### 4.2. VineSens Architecture

#### 4.2.1. Global Overview

**Figure 2:**
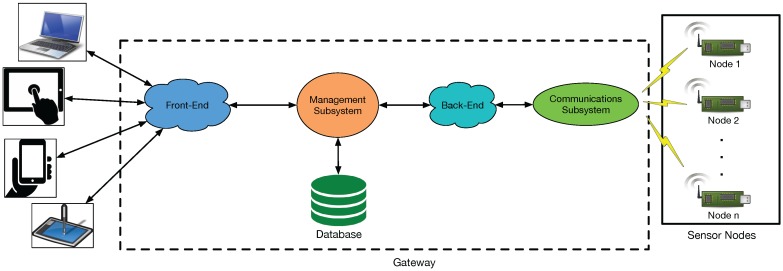
Global overview of the system.

Figure 2 shows an overview of the VineSens architecture, including its main components and how they are connected. Sensor nodes are in Figure 2 on the right. Their objective is to collect sensor data and send them to the gateway through a wireless interface to be processed.

The gateway is composed by two main subsystems:

- Communications subsystem. It is responsible for transferring the information collected from the sensor nodes to the management subsystem. Communications are performed through a Representational State Transfer (REST) Application Programming Interface (API).
- Management subsystem. This subsystem is divided into two parts. On the one hand, it provides a back-end server to manage the data obtained through the communications subsystem. On the other hand, it provides a front-end server to offer access to VineSens to remote users. It also manages a database that stores the collected data and all the necessary data structures of the back-end and the front-end.

#### 4.2.2. Sensor Nodes

Sensor nodes are composed by the hardware required to collect measurements on the environmental conditions. Each node is controlled by an ESP8266, which is a wireless low-cost and low-power System-on-Chip (SoC) that supports IEEE 802.11b/g/n at 2.4 GHz. It contains an integrated TCP/IP (IPv4) protocol stack and includes WPA/WPA2 authentication. Its programming can be performed through the Arduino Integrated Development Environment (IDE) and making use of sensor libraries already provided by the Arduino community.

**Figure 3:**
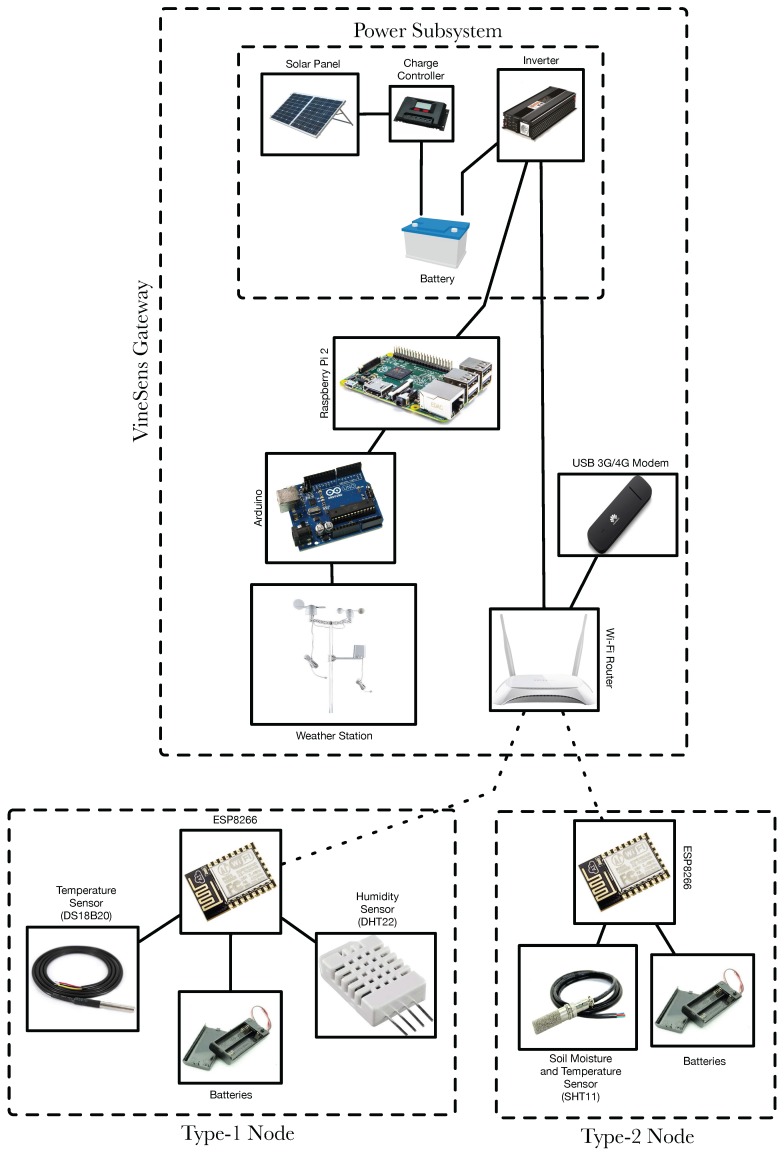
VineSens’ hardware components.

**Figure 4:**
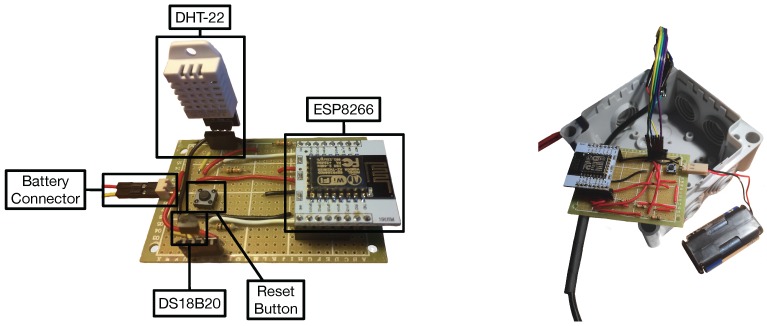
Type-1 node out of the box (**left**) and encapsulated (**right**).

As it can observed in Figure 3 at the bottom, two types of nodes were created. Type-1 nodes monitor atmospheric data: ambient temperature and relative humidity. For such a purpose they use a DS18B20 (Maxim Integrated, San José, CA, USA) and a DHT22 (AM2302, Aosong Electronics Co. Ltd., Guangzhou, China). Note that, in some scenarios (for instance, indoors), it would be easy to use the DHT22 both as a relative humidity and temperature sensor. However, as it is detailed in Section 5.1, this sensor requires an external protection (a Stevenson screen) that reduces flexibility when placing the sensor next to a plant. The DS18B20 is already encapsulated and does not need additional protection, so it can be placed easier than the DHT22 in the preferred location of the plant, what makes it a better choice for measuring temperature. Figure 4 shows the final prototype of a Type-1 node.

**Figure 5:**
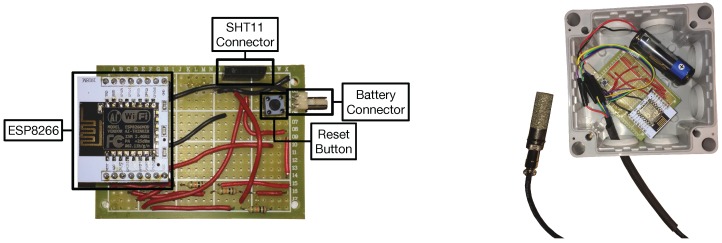
Type-2 node out of the box (**left**) and encapsulated (**right**).

Type-2 nodes collect data from the soil. They use SHT11 (Sensirion AG, Staefa, Switzerland) sensors for obtaining temperature and moisture. It is important to emphasize that SHT11 cannot be used directly to measure soil parameters, but some vendors [48] have encapsulated it in a washable metal mesh (shown on the right in Figure 5) that ensures good corrosion resistance. Additionally, each node is encapsulated in an IP66 waterproof case [49], whose dimensions are 100 mm × 100 mm × 55 mm.

**Figure 6:**
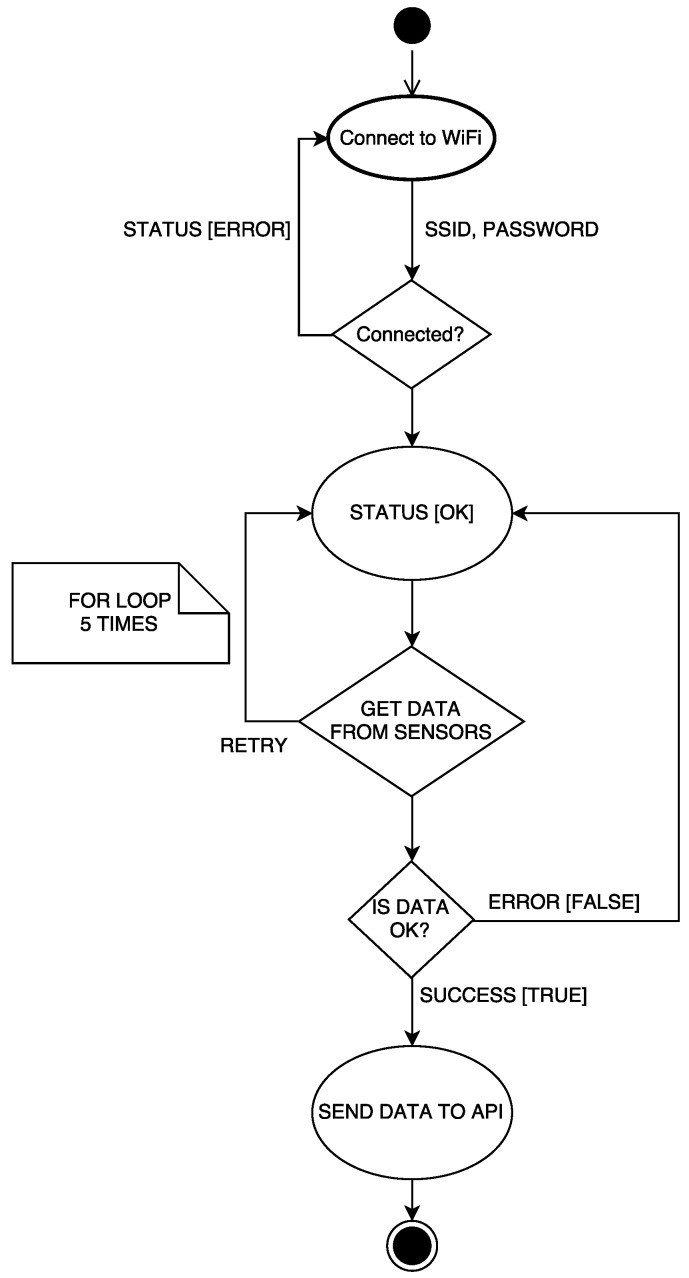
ESP8266 activity diagram for the sensor data collection.

Sensor data collection is performed through the software implemented into an ESP8266 (Espressif Systems, Shanghai, China). It has been designed to optimize the duration of the battery. Thus, a sensing node wakes up from deep sleep mode and tries to connect to the Wi-Fi network. If the connection is successful, the node obtains the data from the sensors and sends them using the REST API. Then, the node goes back to deep-sleep mode for the next hour. This process is illustrated in the activity diagram shown in Figure 6.

#### 4.2.3. Gateway Hardware

##### Gateway Control Hardware

The requirements of the management subsystem have to be provided through a central node that has to offer better performance than the sensor nodes of the system, since it has to process simultaneously the data coming from the sensors and the requests from the remote users. To develop and implement this subsystem it can be used either a dedicated server (i.e., a desktop computer or a laptop), or a Single-Board Computer (SBC), which is a platform that in its most basic format integrates a SoC, RAM, and a number of ports on a reduced form-factor. It was chosen the latter, since it reduces considerably the cost, size, and lowers the consumption, facilitating the integration of the server in an electrical installation without too many complications, due to its low-power requirements and heat dissipation.

Over the last years, many SBCs have been presented, like the Raspberry Pi 1, 2, and 3 [50,51,52], the HummingBoard [53], the BeagleBone [54], the Banana Pi [55], and many others. We selected among them the Raspberry Pi 2 Model B, because it offers a good trade-off between cost (around $50 as of writing), size (only 85.6 mm × 53.98 mm), and performance. It sports an ARM quad-core Cortex-A7 processor running at 900 MHz, and includes 1 GB of RAM, 4 USB ports, 40 GPIO pins, HDMI output, an Ethernet connector, a 3.5 mm audio jack, and a micro-USB that powers the SBC.

##### Communications Subsystem Technology

As it is illustrated in Figure 2, the communications interfaces connect the sensor nodes scattered throughout the farm to the API-based back-end, which collects the data to store it later in VineSens’ database.

Table 3 contains the most relevant wireless communications technologies that have been previously proposed in the literature to connect the nodes. Among them, it was decided to use the most common Wi-Fi standards (i.e., IEEE 802.11b/g/n) due to their international acceptance, and their ability to work at the Industrial, Scientific and Medical (ISM) band at 2.4 GHz. Moreover, Wi-Fi standards have the capacity to transmit data at enough speed (between 11 Mbit/s and 300 Mbit/s).

It is also necessary to select the device that connects VineSens to the Internet. Since the farm is in a remote location (e.g., there is no electricity or landline), only 3G/4G communications are available. Due to this restriction, it was chosen a TP-LINK router (TL-MR3420, TP-LINK Technologies Co. Ltd., Shenzen, China), which is able to share UMTS/HSPA/EVDO connections with IEEE 802.11n clients at a transfer speed of up to 300 Mbps. Regarding the 3G/4G modem, it was selected a USB HUAWEI modem (E3372S-153, Huawei Technologies Co. Ltd., Shenzen, China), which features LTE Category 4, with downlink and uplink speeds of up to 150 Mbps and 50 Mbps, respectively.

##### Weather Station

For obtaining wind speed, wind direction, and rain it was used a weather station placed at a high spot of the farm. The weather station is composed by three elements: an Arduino [62], a data acquisition board (a Weather Shield from SparkFun (DEV-12081, Sparkfun Electronics, Niwot, CO, USA)), and different sensors. The Weather Shield is really useful, since it allows for obtaining data about the barometric pressure, the relative humidity, the luminosity, and the temperature. It also has connections to attach a weather-vane, a pluviometer, and an anemometer.

##### Gateway Power Subsystem

Since the gateway is located on a farm without access to the grid, it is necessary to provide the system with a self-sustainable, reliable, and constant power subsystem. The traditional, yet most used approach, consists in using batteries. Nevertheless, these energy reservoirs can store a finite amount of energy, and their maintenance is a bottleneck. A technique that circumvents this limitation is environmental energy harvesting that exploits ubiquitous energy sources in the operating space. In the present application, the most feasible harvesting techniques are related to solar and kinetic energy sources (i.e., the usage of a wind turbine or the water from the pipes). Because of its higher power and lower cost, the power subsystem proposed by VineSens is a solar power subsystem that includes the following components:

- A 160 W solar polycrystalline panel with a 12 V output and an internal regulator for the battery. This kind of panel was chosen because it gives a good trade-off between cost, weight, and efficiency.
- A 90 Ah lead-acid battery. This kind of battery would last three successive days without sun, what is considered enough for the place where VineSens was deployed.
- A 300 W power inverter (MJ-300-12, Xunzel, Mendaro, Spain) to convert from 12 V to 220 V. Note that it would be also valid to use a 12 V/5 V DC converter if the Raspberry Pi-based gateway was the only device actually powered, but it was selected the DC/AC inverter to increase the scalability of the system and allow us to add other AC-powered devices easily (like the TP-LINK Wi-Fi router used by the gateway).

The final scheme of the power subsystem is shown at the top in Figure 3.

#### 4.2.4. Gateway Software

The gateway’s management subsystem provides three main services that are run on the Raspberry Pi 2:

- A REST API that monitors and stores the data collected by the sensor nodes and that acts as a back-end, which has been implemented using Node.js [63]. The back-end could be potentially on the cloud, but it is actually on the Raspberry Pi (i.e., on the crop) to be able to access it from the farm without requiring an Internet access.
- A front-end that allows users to visualize the stored data and notifications through a web application.
- A data validation service and a mechanism for notifying alerts.

Regarding the REST API, it offers the following main methods:

1. /add/type1/:node. GET and POST requests that are used to manage Type-1 node data. It supports the following key-value pairs:
  (a) temp: temperature.
  (b) hum: relative humidity.
  (c) volt: voltage.
2. /add/type2/:node. GET and POST requests. Similar to the previous request, but for Type-2 nodes (it supports the same key-value pairs as the previous Type-1 node request).
3. /add/weather-station. GET and POST requests. It is used for storing data related to the weather station. It supports the following key-value pairs:
  (a) rain: accumulated rain in L/$m^{2}$.
  (b) wind: wind speed in km/h.
  (c) dirWind: direction of the wind in degrees.
4. /nodes/:node. GET request. It allows for obtaining the data of one specific node. It only supports one key-value pair:
  (a) limit: number of results to be shown.

**Figure 7:**
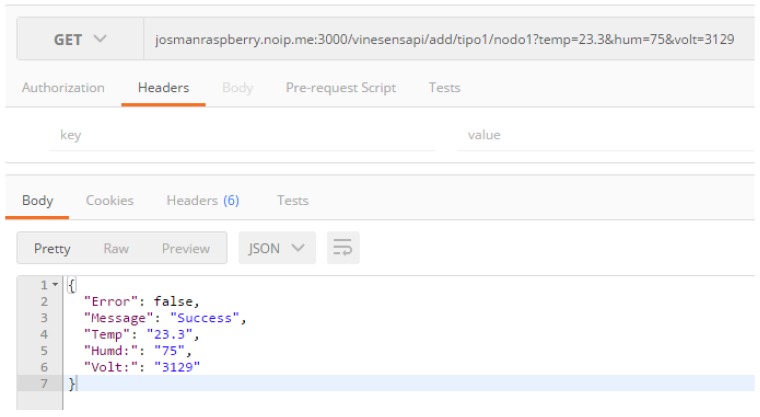
Example of the execution of an API store request.

Figure 7 shows an example of an API request and its result.

Regarding the storage of the collected data, a MySQL database was selected, since it is widely used and there is a strong user community behind it. Moreover, the back-end and front-end servers chosen were compatible with such a database.

The front-end is responsible for controlling and managing the interaction with the users. Multiple technologies were used: PHP, HTML5, CSS3, JavaScript, JQuery, AJAX, JSON, and Bootstrap 3. It was developed according to the Responsive Web Design [64], a design philosophy whose objective is to adapt the appearance of a web page depending on the device used to browse it. Therefore, the web application can be used in multiple devices, like PCs, MACs, tablets, or smartphones.

The third service provided by the gateway is the alert service. Such a service is implemented in Linux and PHP. Every hour the server checks if there are new alerts and sends them to the user.

## 5. Results

### 5.1. Sensor Network Deployment

**Figure 8:**
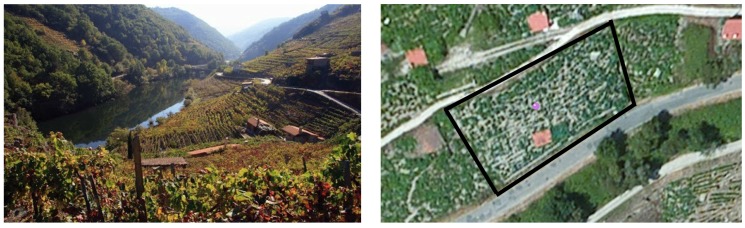
Area where VineSens was deployed (**left**) and map location (**right**) (Sources: Vivirgaliciaturismo [66] under CC License, ©2017 Google).

**Figure 9:**
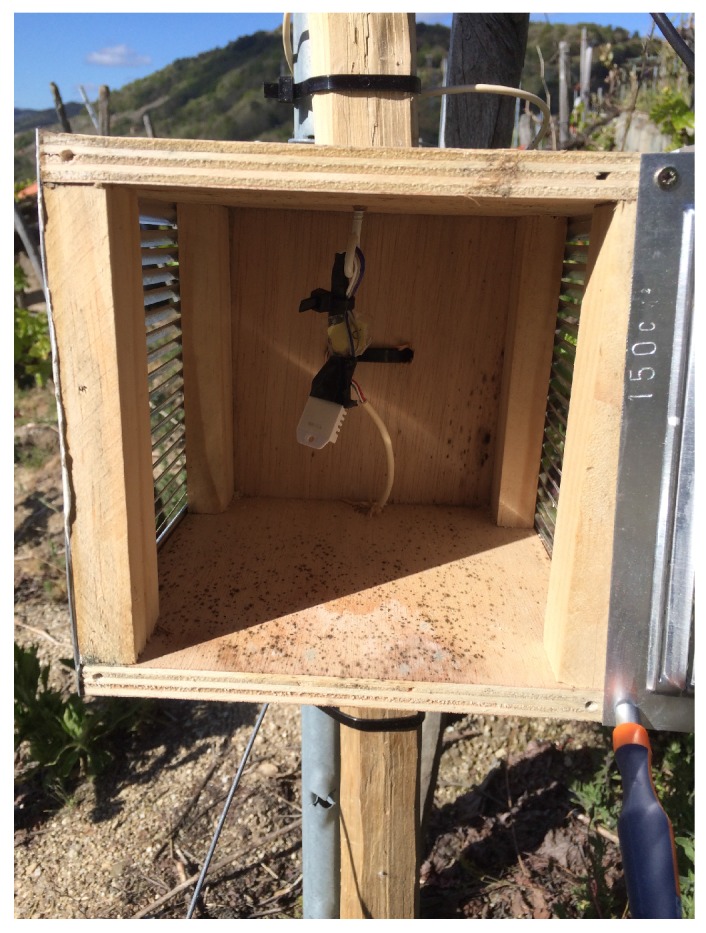
Relative humidity sensor inside Stevenson screen.

The deployment of VineSens was carried out in a 0.14 ha (0.34 acres) vineyard located in Ribeira Sacra (Galicia, Spain). Its shape and inclination can be observed in Figure 8. Four nodes were deployed in this vineyard: two Type-1 nodes and two Type-2. Since there are two types of nodes that collect different data, two different strategic locations of the farm were selected in order to obtain an accurate representation of the environment. Because the relative humidity sensor of Type-1 nodes is not protected against the weather, such nodes were encapsulated in Stevenson screens [65]. In this way, such a kind of node is isolated from the rain, but the air comes through the grille, allowing for measuring relative humidity. Figure 9 shows the interior of one of the Stevenson screens, where the sensor is placed at the center.

**Figure 10:**
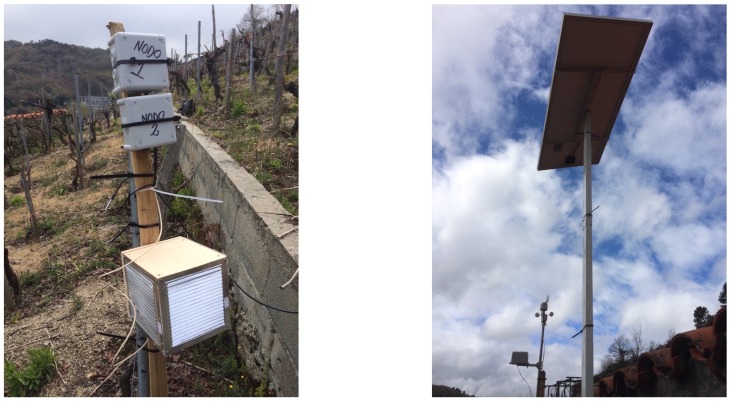
Example of Type-1 and Type-2 nodes deployed on the vineyard (**left**) and galvanized structure for the solar panel (**right**).

Regarding the temperature sensors of the Type-1 nodes, they were located one meter above the ground. The relative humidity sensors inside the Stevenson screen were 30 cm from the soil. Both temperature and moisture sensors of the Type-2 nodes were placed 15 cm underground. The final disposition of one pair of nodes is shown in Figure 10 on the left. The solar panel that powers the gateway was placed on a galvanized steel structure (in Figure 10, on the right) that was erected next to the farm’s wine cellar. In Figure 10, in the background, the weather station can be observed.

### 5.2. Data Visualization

In order to analyze the data collected from the vineyard, the user has to authenticate in VineSens’ web application. Once logged in, the user selects the data to visualize (ambient or soil data) during a certain date range. There are different ways to display the data: graphic curves (daily or hourly average), raw data tables, or a summary that compares the data among the different nodes.

**Figure 11:**
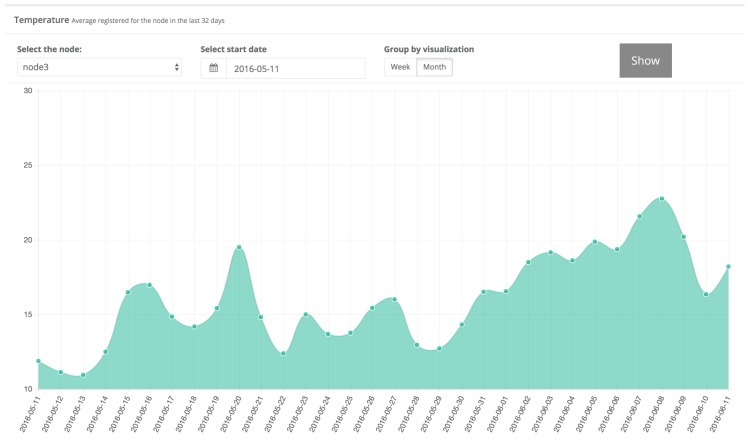
Average daily temperature values collected by node 3.

**Figure 12:**
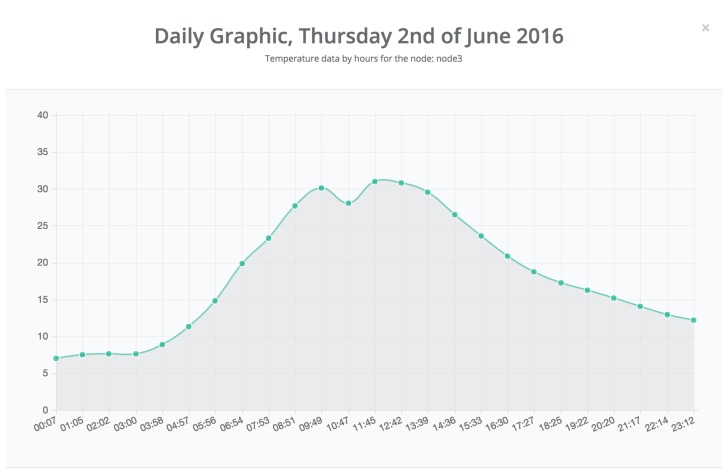
Temperature values collected by node 3 on an hourly basis.

Figure 11 shows a snapshot of the data obtained by node 3 between 11 May and 11 June 2016. Clicking on a specific day shows the hourly measurements. As an example, Figure 12 shows the hourly temperature obtained by node 3 on 2 June 2016.

**Figure 13:**
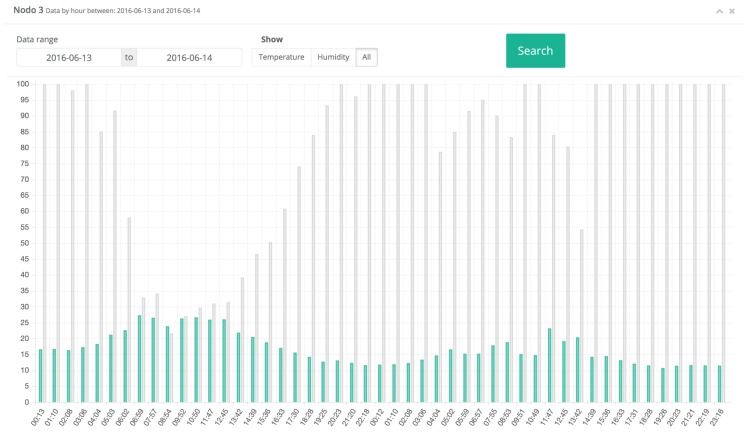
Data collected on node 3 on 13–14 June 2016.

**Figure 14:**
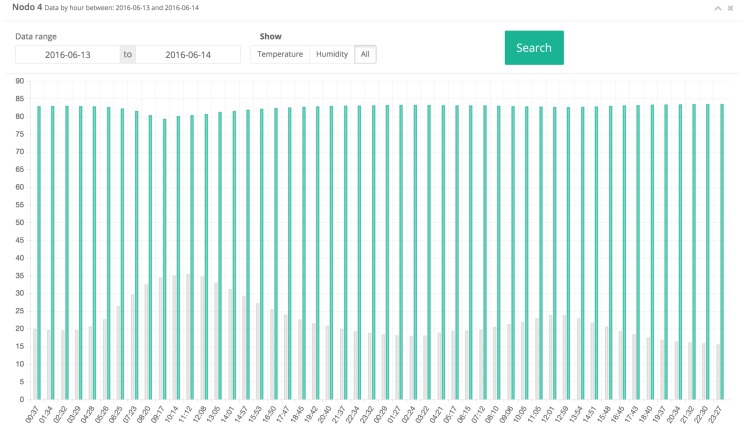
Data collected on node 4 on 13–14 June 2016.

Figure 13 and Figure 14 show a comparison of the relative humidity, soil moisture, and temperature values obtained by nodes 3 and 4 (they are Type-1 and Type 2 nodes, respectively). It can be observed that, although initially data may seem similar, fine variations can alter the development and necessities of the vine. For instance, it can be observed the oscillations on the relative humidity measured by node 3, which were due to the rain, and a progressive decrease on the temperature. However, note in Figure 14 (i.e., soil parameters measured by node 4 for the same day) how soil acts as a buffer, retaining moisture from the rain and a relatively high temperature.

### 5.3. Alert Notifications

VineSens manages three different types of alerts:

- Downy mildew alerts.
- Power alerts. It warns about energy outages on the nodes.
- Ambient temperature alerts.

**Figure 15:**
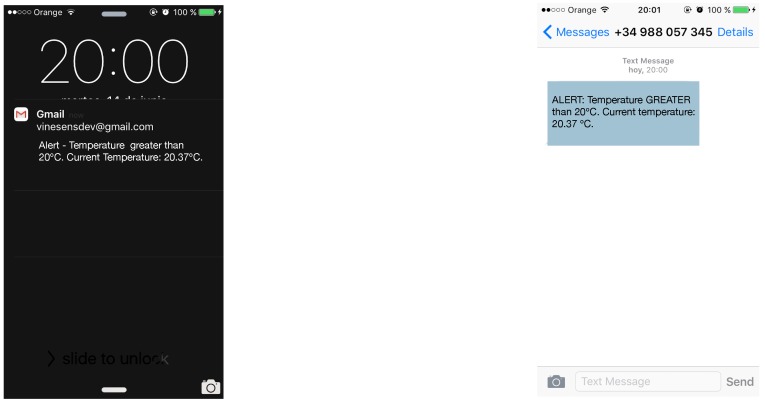
Alerts received through e-mail (**left**) and SMS (**right**).

Once an alert is enabled and its parameters are configured, it is checked once per hour. If the parameters meet the alert criteria, a message will be displayed on the web interface and an SMS or e-mail will be sent to the user. Figure 15 shows an example of a notification sent by e-mail and SMS of a temperature alert.

**Figure 16:**
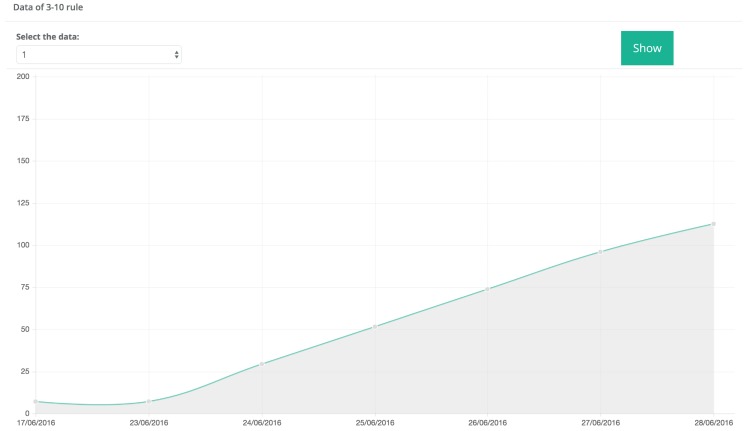
Evolution of the downy mildew development index.

Regarding downy mildew alerts, by default, the system monitors the disease development index until it reaches 90%. In that moment, the system sends a warning alert. When the index reaches 100%, the system sends an urgent alert. An example of the monitoring of the downy mildew development index is shown in Figure 16. In such a Figure it can be observed that, since the daily development percentages shown in Table 1 are not unitary increments, the percentage can rise over 100% from one day to the next one. After applying downy mildew treatments, the alert has to be re-enabled: this is the way the user indicates that the treatments have been applied, so downy mildew index values are re-started.

### 5.4. Weather Station

**Figure 17:**
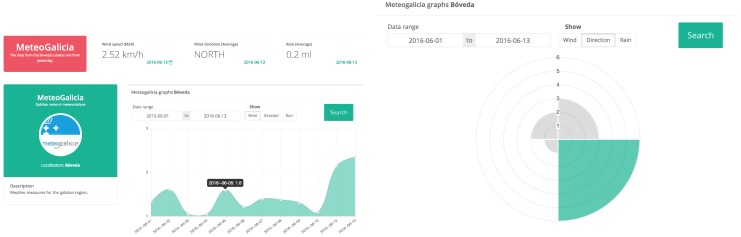
Wind speed (**left**) and direction (**right**) read by the weather station on 1–13 June 2016.

VineSens’ web application offers a specific section for visualizing the data collected through the weather station. Once a day, the data on wind speed, wind direction, and rain are collected from the weather station. Figure 17 shows a detailed view on the wind speed and direction for the first two weeks of June of 2016.

### 5.5. Node Energy Consumption

In order to determine the number of days that the nodes will last with batteries, their energy consumption was obtained. Note that a node is basically made of an ESP8266 and its respective sensors, whose amount and type depend on the kind of node. Table 4 shows the current consumption of each type of node when transmitting data from the sensors embedded and when in deep-sleep mode. The average hourly and daily consumption of both types of nodes is shown in Table 5. In such a Table it can be observed that the different battery packs allow the system to last between 326 and 816 days on a full discharge. However, note that such estimated duration figures are theoretical, based on the whole charge and discharge of the battery. In practice, since the ESP8266 powering voltage ranges from 2.8 V and 3.5 V, once the batteries are below 2.8 V, the ESP8266 will stop working properly. From the experiments performed, it was determined that the actual duration of the batteries was between a month and a half, and three months. This fact indicates that, in order to get a longer lasting system, other powering systems would have to be considered (for instance, a low-voltage solar power system).

### 5.6. Phytosanitary tReatment Use

In the area where VineSens was deployed, the preventive measures start to be applied around April and May, and they end in August. Grape harvest usually occurs around 15th–20th September. During these months, phytosanitary treatments are commonly applied every 12–15 days. If the weather suddenly varies from very rainy to dry in a short amount of time (the ideal conditions for the development of downy mildew), then it is recommended to apply the treatments every 5–7 days. Such treatments consist in applying copper sulfate, which is successful for controlling the disease, but it can damage the microorganisms in the soil and insects, and it is considered moderately toxic for humans and mammals. Therefore, the treatment should be used sparingly and with caution.

Table 6 and Table 7 compare traditional spraying planning methods with VineSens in terms of the number of sprayings that would be actually necessary to prevent downy mildew. The methods included in such Tables are:

- Spraying according to phytosanitary warnings. Every week the CSIC (Spanish National Research Council) publishes online recommendations for the vine growers on the treatments to be performed. In particular, the CSIC has control vineyards in an area that is 80 km from the place where VineSens was deployed, so warnings can be considered valid since weather in both areas is similar.
- Traditional spraying. It consists in following the spraying recommendations previously mentioned: farmers have to apply treatments every 12–15 days from April–May and, during the period with the highest risk, they should reduce the spraying period to 5–7 days. In Table 6 and Table 7 two variants of traditional spraying are considered: conservative and relaxed. The conservative approach tends to spray as much as possible according to the traditional schedule, while the relaxed approach tends to spray the least, but respecting CSIC recommendations.
- VineSens recommendations. VineSens algorithms estimate spraying by calculating the downy mildew development index. When such an index is high enough, VineSens sends a warning to the farmer, who sprays and resets the index. Two versions are illustrated to compare with the other systems: a conservative approach that suggests spraying when the downy mildew development index reaches 80 %, and a more relaxed approach that waits until the index is greater than 90 %.

It can be observed from Table 6 and Table 7 that the number of doses varies depending on the spraying method, but it ranges from 11 to 13 doses. In terms of economic cost, it may not seem a great difference, but it depends on the size of the vineyard. For instance, the cost of the products applied on the farm monitored (copper sulphate and Mikal Plus) during a season were, on average, €108.8 (including the products, labor, and machinery). The farmer usually applied approximately 15 doses, so the cost per dose can be estimated around €7.25. Assuming such a cost per dose, it can be concluded that the use VineSens for the period analyzed in 2016 would derive into savings of up to €14.5. Nonetheless, note that it is just a small farm (half an acre): according to the Spanish Statistical Office (INE [67]) the average vineyard surface in Spain is 31.54 ha (77.93 acres), which means that the average savings in the selected area and during the year 2016 would reach on the average farm €2260. It must be noted that these differences are not greater due to the specific weather conditions of the last season, which have been especially favorable for downy mildew development. In fact, according to the local agricultural union estimations [68], in the area where VineSens was deployed, downy mildew proliferation reduced the grape harvest by 12 % (the reduction estimated for nearby areas ranged between 20 % and 28 %).

## 6. Conclusions

This article presented VineSens, a platform based on wireless sensor nodes that allows for remote monitoring vineyards. Specifically, VineSens supports decision-making to control downy mildew, a pseudo-fungus that is one of the most feared diseases by vine growers. After reviewing the most popular epidemiological models, it was decided to focus on the Rule 3-10, which allows VineSens to propose a custom treatment plan for downy mildew. The system notifies when the disease has reached a threshold when preventive measures have to be taken. Thus, the system helps to avoid the excessive use of pesticides and herbicides, reducing the impact in the environment.

VineSens was deployed for the first time in Ribeira Sacra (Galicia, Spain) in February 2016. The deployment included two types of nodes based on ESP8266 microcontrollers. Type-1 nodes monitor atmospheric data and Type-2, soil parameters. The nodes were designed to minimize energy consumption and were encapsulated into protective IP66 boxes. A solar power subsystem was designed to fulfill the power requirements of the farm in an ecologically sustainable way.

The information collected by VineSens is presented through a user-friendly web portal for management and visualization. The web-based system can be accessed from any computer, tablet or smartphone with the only requirement of having a browser and an Internet connection. Furthermore, the system provides up-to-date information for managing the vineyard in the form of alerts via SMS and e-mail.

Regarding the experiments performed, they verified that the system worked as expected in a real-world scenario and allowed for accessing remotely the data collected on the weather, the nodes, and the alerts associated with the development of downy mildew. The energy consumption of the sensor nodes was also measured and it was concluded that, with four AA batteries, the system was able to last up to three months. Finally, the savings related to the reduction of the phytosanitary treatments applied were estimated and it was concluded that, in an average farm, substantial savings can be achieved.

To sum up, all results obtained confirm that VineSens provides a good tool for vine growers to remotely manage the state of a vineyard and to automate the detection of diseases like downy mildew. Such a detection not only generates savings for the farmer, but also protects the environment and helps to create more sustainable vineyards.

## Figures and Tables

**Table 1:** Goidanich’s table for following the development of downy mildew.

Average Temperature (°C)		Daily Development (%) RH < 75%	Daily Development (%) RH ≥ 75%	Average Temperature (°C)		Daily Development (%) RH < 75%	Daily Development (%) RH ≥ 75%
12.	0	0	5.25	19.	0	12.5	16.6
	25	4.4	5.75		25	12.9	17.5
	50	4.7	6.2		50	13.4	18.3
	75	5	6.7		75	13.7	19.3
13.	0	5.3	7.1	20.	0	14.2	20
	25	5.7	7.7		25	14.5	20.5
	50	6	8		50	14.8	21
	75	6.4	8.5		75	15	21.5
14.	0	6.6	9	21.	0	15.3	22.2
	25	6.8	9.4		25	15.7	22.2
	50	7.1	9.7		50	16	22.2
	75	7.3	10.2		75	16.3	22.2
15.	0	7.6	10.6	22.	0	16.6	22.2
	25	7.8	10.8		25	17	22.2
	50	8.1	11.1		50	17.3	23.5
	75	8.3	11.3		75	17.7	24.4
16.	0	8.5	11.7	23.	0	18.1	25
	25	9	12		25	18.1	25
	50	6.3	12.5		50	18.1	25
	75	9.6	12.9		75	18.1	25
17.	0	10	13.25	24.	0	18.1	25
	25	10.3	13.6		25	17.7	24.3
	50	10.5	14.3		50	17.3	23.5
	75	10.75	14.75		75	17	23.2
18.	0	11.1	15.3	25.	0	16.6	22.2
	25	11.48	15.2				
	50	11.7	16				
	75	12.1	16.3				

**Table 2:** Main characteristics of the different epidemiological models for preventing downy mildew.

Model	Type	Advantages	Drawbacks
Rule 3-10 (Baldacci, 1947)	Empirical	Well-known, simplicity	The model does not take oospore maturing into account and does not distinguish among the different stages of the infection process
EPI model (Stryzik, 1983)	Empirical	Good trade-off between complexity and performance	False negatives, it requires the weather record
DMCast (Park et al., 1997)	Empirical	Good trade-off between complexity and performance	False negatives
UCSC (Rossi et al., 2008)	Mechanistic	It does not require calibration or correction and provides an accurate, detailed, and dynamic simulation of the sexual stage	Very complex

**Table 3:** Wireless communications technologies proposed for monitoring vineyards.

Technology	Frequency Band	Data rate	Range	Power	Battery Operation	Nodes
Wi-Fi/IEEE 802.11b/g/n [30,56]	2.4 GHz, 5.8 GHz	11 - 105 Mbit/s	10-100 m	High	Rechargeable (hours)	32
ZigBee/IEEE 802.15.4 [32,33]	868 MHz, 2.4 GHz	250 kbit/s	10-300 m	Very low	Alkaline (months to years)	65000
Bluetooth/IEEE 802.15.1 [57,58]	2.4 GHz	723 kbit/s	10 m	Low	Rechargeable (days to weeks)	8
UWB/IEEE 802.15.3a	3.1-10.6 GHz	>110 Mbit/s	4-20 m	Low	Rechargeable (hours to days)	128
DASH7/ISO 18000-7 [59]	433 MHz	27.8 kbit/s	250 m	Very Low	Alkaline (months to years)	Many
Z-Wave	900 MHz	40 kbit/s	100 m	Very Low	Alkaline (months to years)	232
6LowPAN	2.4 GHz	200 kbit/s	200 m	Very Low	Alkaline (months to years)	100
RFID [60,61]	30 KHz-3 GHz	<640 kbit/s	1 cm-10 m	Very Low	Alkaline (months to years)	Many

**Table 4:** Current consumption of each type of node during transmission and in deep-sleep mode.

Node Type	Sensors	Consumption	
Type 2	SHT11	Transmission	93.5 mA
		Deep-sleep	6μA
Type 1	DS18B20, DHT22	Transmission	75 mA
		Deep-sleep	6μA

**Table 5:** Total consumption and duration for each type of node.

Node	Total per Hour	Total per Day	Batteries	Estimated Duration (Full Discharge)	Actual Duration
Type 2	0.266 mA/h	6.384 mA	2 × 2100 mAh AA batteries in series	328 days	93 days
			4 × 2100 mAh AA batteries in parallel (2 to 2)	657 days	182 days
Type 1	0.214 mA/h	5.143 mA	2 × 2100 mAh AA batteries in series	408 days	93 days
			4 × 2100 mAh AA batteries in parallel (2 to 2)	816 days	182 days

**Table 6:** Traditional downy mildew prevention methods versus VineSens (first part).

	Phytosanitary Warning		Traditional (Conservative)		Traditional (Relaxed)		VineSens (80%)			VineSens (90%)		
**Date**	**Event**	**#Dose**	**Event**	**#Dose**	**Event**	**#Dose**	**Event**	**Downy Mildew Index**	**#Dose**	**Event**	**Downy Mildew Index**	**#Dose**
03/01/16							Start monitoring.	0		Start monitoring.	0	
03/28/16	Oosphore maturation detected due to high temperature during winter.							0			0	
04/01/16			Beginning of the treatment.	1				0			0	
04/15/16			Next dose.	2				0			0	
04/19/16							Detected favorable development conditions.	5.75		Detected favorable development conditions.	5.75	
04/20/16	First clear symptoms manifested in the farms monitored. Beginning of the treatment recommended.	1						11.05			11.05	
04/29/16	Cool nights are slowing the development of the disease. Remain vigilant.							16.8			16.8	
05/01/16			Next dose.	3	Begining of the treatment.	1		16.8			16.8	
05/06/16	Increased risk of outbreaks due to high nocturnal temperatures and the increase in RH.	2						55.9			55.9	
05/13/16	Increased risk, but treatment is only needed on vines that show obvious signs.	Optional						55.9			55.9	
05/15/16			Next dose.	4	Next dose.	2		66.1			66.1	
05/17/16							Downy mildew alert: index over 80%.	81.0	1		81.0	
05/18/16							Treatment applied.Index reset.	6.2			87.2	
05/19/16										Downy mildew alert: index over 90%.	97.4	1
05/20/16	A new dose of the treatment should be applied due to the beginning of the flowering.	3						27.88		Treatment applied. Index reset.	11.48	
05/27/16	Weather is favoring the development of the disease, but only the vines already infected should be treated.	Optional						79.88			62.98	
05/28/16							Downy mildew alert: index over 80%.	84.63	2		68.23	
06/01/16			Next dose.	5	Next dose.	3		26.35		Downy mildew alert: index over 90%.	94.58	2
06/03/16	Increased risk of infection. A new dose of the treatment should be applied.	4						49.35			23.0	
06/07/16							Downy mildew alert: index over 80%.	95.15	3		68.8	
06/09/16										Downy mildew alert: index over 90%.	99.5	3
06/10/16	High risk of infection. Renew the dose if the treatment was not successful.	Optional	Next dose (high risk).	6	Next dose (high risk).	4		39.0			8.3	
06/14/16							Downy mildew alert: index over 80%.	82.78	4		52.08	
06/17/16	Numerous farmers have reported damage in their vineyards. Renew the dose.	5	Next dose (high risk).	7	Next dose (high risk).	5		11.45			63.53	

**Table 7:** Traditional downy mildew prevention methods versus VineSens (second part).

	Phytosanitary Warning		Traditional (Conservative)		Traditional (Relaxed)		VineSens (80%)			VineSens (90%)		
**Date**	**Event**	**#Dose**	**Event**	**#Dose**	**Event**	**#Dose**	**Event**	**Downy Mildew Index**	**#Dose**	**Event**	**Downy Mildew Index**	**#Dose**
06/20/16										Downy mildew alert: index over 90%.	98.43	4
06/22/16							Downy mildew alert: index over 80%.	88.85	5		42.5	
06/23/16	The high risk of infection remains. Renew the dose on plants already showing clear damage.	Optional	Next dose (high risk).	8	Next dose (high risk).	6		21.0			63.5	
06/26/16										Downy mildew alert: index over 90%.	91.4	5
06/29/16							Downy mildew alert: index over 80%.	87.8	6		38.9	
07/01/16	[Missing report due to the IT problems in the phytosanitary news server]		Next dose (high risk).	9	Next dose (high risk).	7		23.58			62.48	
07/04/16										Downy mildew alert: index over 90%.	104.98	6
07/05/16							Downy mildew alert: index over 80%.	87.08	7		21.0	
07/08/16	High risk continuous. Renew the treatment.	6	Next dose (high risk).	10	Next dose (high risk).	8		55.1			76.1	
07/09/16										Downy mildew alert: index over 90%.	93.4	7
07/10/16							Downy mildew alert: index over 80%.	88.7	8		16.3	
07/15/16	Optimal infection conditions. Renew the treatment.	7	Next dose (high risk).	11	Next dose (high risk).	9		52.5			68.8	
07/17/16										Downy mildew alert: index over 90%.	103.5	8
07/19/16							Downy mildew alert: index over 80%.	87.2	9		33.6	
07/22/16	Slight decrease in risk of infection, but it is still high. Apply only the treatment to infected plants.	Optional	Next dose (high risk).	12	Next dose (high risk).	10		47.0			73.3	
07/23/16							Downy mildew alert: index over 80%.	86.7	10		86.7	
07/24/16										Downy mildew alert: index over 90%.	102.4	9
07/29/16			Final dose.	13	Final dose.	11	Downy mildew alert: index over 80%.	91.7	11		76.0	
07/30/16										Downy mildew alert: index over 90%.	91.3	10
07/04/16							Downy mildew alert: index over 80%.	85.7	12		70.4	
08/05/16	The risk remains low. No treatment should be applied except in very specific situations.							13.4			83.8	
08/06/16										Downy mildew alert: index over 90%.	100.4	11
08/10/16							Downy mildew alert: index over 80%.	89.3	13			
08/12/16	In most areas, bunches are already insensible to the disease. No further actions need to be taken.											

## Abbreviations

The following abbreviations are used in this manuscript:

API: Application Programming Interface
CSIC: Spanish National Research Council
DSS: Decision Support System
GHG: Greenhouse Gas
IDE: Integrated Development Environment
ISM: Industrial, Scientific and Medical
LAI: Leaf Area Index
REST: Representational State Transfer
RH: Relative Humidity
RPAS: Remotely Piloted Aerial Systems
SBC: Single-Board Computer
SoC: System-on-Chip
WSN: Wireless Sensor Network
